# 我国云南曲靖肺癌高发区环境流行病学调查研究

**DOI:** 10.3779/j.issn.1009-3419.2012.03.05

**Published:** 2012-03-20

**Authors:** 霖琳 张, 继华 李, 亚杰 汪, 国平 吴, 复盛 魏

**Affiliations:** 1 100012 北京，中国环境监测总站 National Environmental Monitoring Center, Beijing 100012, China; 2 655000 曲靖，曲靖市疾病预防控制中心 Qujing Centers for Disease Control and Prevention, Qujing 655000, China; 3 100055 北京，中国高新投资集团公司 China Gao Xin Investment Group Corporation, Beijing 100055, China

**Keywords:** 肺肿瘤, 环境污染, 煤, 流行病学, Lung neoplasms, Environmental pollution, Coal, Epidemiology

## Abstract

**背景与目的:**

我国云南省东北部的曲靖是全世界肺癌高发地区之一，当地居民长期暴露在燃煤烟气中，本研究旨在全面了解我国云南曲靖肺癌高发区的环境背景和污染情况，为肺癌病因学研究提供科学依据，同时也为改善环境状况提供数据支持。

**方法:**

采用多阶段、分层、整群、概率抽样方法随机抽取280个自然村，了解当地自然村使用燃料的种类、附近有无炼焦厂、铁锌厂、化工厂等环境背景和污染情况，并采用二项*Logistic*回归分析方法对调查因素进行分析。

**结果:**

高发区78.1%的自然村使用烟煤和焦煤，低发区78.8%的自然村使用无烟煤和木柴。二项*Logistic*回归分析方法显示，上述因素中仅有燃料种类进入方程（*P* < 0.05），烟煤和焦煤的使用与肺癌高发具有正关联作用，而使用木柴、无烟煤不会对肺癌的高发产生影响。

**结论:**

当地居民使用燃料类型是影响肺癌高发的重要因素，其中烟煤和焦煤对当地肺癌高发有促进作用，而无烟煤或者木柴作用不大或有抑制作用。

## Abstract (en)

**Background and objective:**

Qujing, located in Southwest China, is an area with an extremely high lung cancer incidence. Combustion of coal has exposed local people to great health hazards. The aim of this study is to achieve a thorough understanding of the relationship between environmental pollution and the high incidence of lung cancer in Qujing, Yunnan Province, China. The results would provide a scientific basis and support for the etiology of lung cancer, as well as suggestions on improving the environmental conditions in the area.

**Methods:**

A total of 280 rural villages were selected through stratified cluster random sampling. Environmental background and pollution were investigated, including details on fuel type, coking plant, metal smelting, and chemical plant, among others. *Logistic* regression analysis was used to analyze the investigated factors.

**Results:**

Out of the total number of local villages studied, 78.1% of those with high incidence often use smoky coal and coking. On the other hand, 78.8% of the low-incidence areas use smokeless coal or wood. *Logistic* regression analysis indicated that the coal type used for everyday life was a main risk factor related to lung cancer (*P* < 0.05). Using smoky and coking coals create an alarmingly high risk for developing lung cancer. Meanwhile, smokeless coals and wood seemed to have no significant relationship to the lung cancer incidence.

**Conclusion:**

The fuel type used for everyday life is an important factor in the high incidence of lung cancer in Qujing. Evidently, the use of smoky coal and coke increased the incidence of lung cancer, whereas smokeless coal and wood seem to bring about the contrary.

我国云南省东北部的曲靖是全世界肺癌高发地区之一，当地居民以烟煤为燃料，用不通风的炉灶做饭、取暖，烟气无法直排到室外，造成室内空气的严重污染^[1]^。研究^[2]^表明长期暴露在燃煤烟气中是导致两地居民肺癌持续高发的主要原因，但是近年来随着改炉改灶的进行和社会主义新农村建设，当地环境污染有了明显的改善，但是肺癌发病率和死亡率仍然居高不下^[3]^。2007年在云南省卫生厅的组织下，曲靖地区开展了5万人的大规模肺癌流行病学调查。本研究仅对此次调查过程中环境状况与肺癌发病的部分内容展开讨论，旨在为当地肺癌高发的病因学研究提供科学依据和数据支持。

## 1 材料与方法

### 1.1 调查对象和内容

通过收集历史资料，了解云南曲靖市宣威、富源等地的环境背景情况，包括大气、土壤、水等。采用流行病学问卷调查的方式，掌握当地自然村使用燃料的种类、附近有无炼焦厂、铁锌厂、化工厂等情况，自然村环境污染状况的调查问卷设计见附表。

### 1.2 抽样方法

样本量估算：由于肿瘤发生属于低概率事件，肿瘤的分布呈*Poisson*分布，样本量估计依据*Poisson*分布期望值可信限表^[4]^和滇东（产）燃煤区肺癌发病率的估计值（50/10万）推算，总样本量约50, 000人。

乡级样本：根据肺癌历史发病、死亡情况把曲靖市辖区内产（燃）煤乡镇分为肺癌高发区、次高发区、中发区和低发区。发病率或死亡率≥80/10万为高发区，次高发区50/1万-79/10万，中发区20/10万-49/10万，低发区 < 20/10万。无发病率或死亡率资料的乡镇以2003年-2006年当地县、市级主要医院新诊断的肺癌病例数为依据划分：高发区≥90例、次高发区60例-89例、中等发病区30例-59例和低发区 < 30例。按比例（2:1）通过电脑软件随机抽取18个乡级样本。村级样本：从每个乡级样本随机1个-2个行政村，共抽取28个行政村。调查点：每个村级样本随机抽取居住人口比较集中的6个-8个自然村作为整群抽样调查点，以满足2, 500人-3, 000人的乡级样本量要求，共计280个调查点。

### 1.3 数据分析

用Access建立数据库，将问卷中的信息录入到编好的程序中，把所有汇总后的数据导入到SPSS 18.0软件中进行统计分析，利用*Logistic*回归方法分析变量之间的关系并进行预测。

### 1.4 质量保证和质量控制

本次流行病学调查规模较大，采用了系统、严格的质量控制和保证措施来确保数据的准确性和可靠性，具体做法如下：①组织保障：成立市级流调领导小组、领导小组办公室、技术指导组（省、市级）、现场流调督导组、临床督导组，各县成立相应的协调小组；②业务培训：由技术指导组制定实施计划以及详细的实施细则，对参加调查的流调人员和临床诊断人员进行技术培训；③现场调查：流调表由市疾病预防控制中心统一印制，市、县督导组于流调开展初期和进展中进行定期、不定期现场督导，及时更正发现的错误。流调现场发现问题及时上报市流调技术指导小组。调查表完成后，由各组审核员完成当天调查表的审核，发现问题或缺项的，及时纠正或补充。审核完成后，审核人员签名确认。经过流调技术指导小组检查验收合格后，采用双录入法进行录入，确保数据录入的准确性。

## 2 结果

### 2.1 区域划分和调查对象

根据肺癌筛查的阳性率将宣威和富源分为A、B、C、D四个地区，调整分层区域的结果见表 1。A为高发区，包括宣威市来宾和西宁；B为次高发区，包括富源县后所、大河、墨红以及麒麟区东山；C为中发区，包括宣威市倘塘、龙潭、格宜、龙场、羊场、田坝以及富源县中安、竹园、营上；D为低发区、麒麟区茨营、罗平县阿岗、师宗县雄壁。

**1:** 调整分层区域肺癌筛查阳性率（1/10万） Positive rate of lung cancer screening in adjusted investigating area (per 100, 000)

Investigating area	Sex	No. of participants	Positive rates for lung cancer screening (per 100, 000)	ASR (95%CI) (per 100, 000)	SE^[5]^ (ASR)
Region A	Male	3, 139	1, 787.85	887.36 (631.64-1, 142.07)	129.14
	Female	4, 120	1, 285.95	819.66 (547.54-1, 090.10)	136.14
	Combined	7, 259	1, 506.80	828.04 (649.47-1, 004.63)	89.28
Region B	Male	4, 936	1, 041.51	645.62 (456.95-834.30)	96.26
	Female	5, 408	816.19	544.18 (356.16-732.20)	95.93
	Combined	10, 344	924.52	592.24 (459.86-724.61)	67.54
Region C	Male	8, 706	771.99	409.7 (304.62-514.78)	53.61
	Female	11, 741	503.11	284.94 (203.10-366.79)	41.76
	Combined	20, 447	617.99	339.68 (274.30-405.05)	33.35
Region D	Male	6, 326	255.60	141.93 (73.46-210.41)	34.94
	Female	8, 457	162.34	100.87 (44.20-157.54)	28.91
	Combined	14, 783	202.25	118.74 (75.22-162.25)	22.20
ASR: age standardized rate. Region A: The highest epidemic region includes Laibin, Xining in Xuanwei county; Region B: The higher epidemic region includes Housuo, Dahe, Mohong in Fuyuan county and Dongshan in Qilin county; Region C: The medium epidemic region includes Tangtang, Longtan, Geyi, Longchang, Yangchang, Tianba in Xuanwei county and Zhong’an, Zhuyuan, Yingshang, Fucun in Fuyuan county; Region D: The light epidemic region includes Ciying in Qilin county, Agang in Luoping county and Xiongbi in Shizong county.					

### 2.2 调查问卷统计结果

2007年调查曲靖市宣威、富源产煤市、县、区，调查点分布与曲靖市煤炭资源地理分布一致，行政村样本地理分布无明显偏倚，共走访19个乡镇28个行政村280个自然村，每个自然村填写一份环境状况调查表，总计收集到280份调查问卷，根据低发区主要产无烟煤或者不产煤，燃料使用无烟煤或者木柴，而其它地区均产烟煤，使用烟煤或者焦煤，因此分成高、低两组，其中高发区、次高发区和中发区共计228份，低发区52份，问卷内容统计的详细结果见表 2。

**2:** 自然村环境状况调查统计结果 Statistics of environmental survey of sampling villages

Item	The high epidemic region (*n*=228)	The light epidemic region (*n*=52)
Villages and towns	16	3
Administrative villages	28	6
Natural villages	228	52
Fuel type		
Smoky coal	153	9
Coke	25	2
Smokeless coal	5	18
Wood	45	23
Coking plant	99	14
Windward	1-15	1
Time (year)	2-46	10-15
Output (ton)	20×10^4^-80×10^4^	40×10^4^-50×10^4^
Leeward	1-20	1-2
Time (year)	2-20	12-15
Output (ton)	60×10^4^-80×10^4^	20×10^4^-80×10^4^
Metal smelting plant	71	12
Windward	1-8	1
Time (year)	1-20	1-20
Output (ton)	0.01×10^4^-3×10^4^	0.01×10^4^-3×10^4^
Leeward	1-5	1
Time (year)	1-10	1
Output (ton)	0.03×10^4^-3×10^4^	0.01×10^4^-3×10^4^
Chemical plant	27	3
Windward	1-4	1
Time (year)	34-38	3
Output (ton)	60×10^4^-300×10^4^	1×10^4^
Leeward	1	0
Time (year)	1	0
Output (ton)	6×10^4^	0

根据表 2中汇总的数据统计得出，高发区78.1%的自然村使用烟煤和焦煤，43.4%自然村附近有焦化厂，31.1%附近有铁锌厂，11.8%附近有化工厂；低发区78.8%的自然村使用无烟煤和不产煤，26.9%自然村附近有焦化厂，23.1%附近有铁锌厂，5.8%附近有化工厂。炼焦厂和铁锌厂的使用年限和产量在高、低发区差异不大，但高发区附近的化工厂较为集中，比低发区的使用年限长且产量较高。高发区的工厂在上风向的较多，对自然村的污染较工厂在下风向的严重。

### 2.3 调查问卷二项*Logistic*回归

由于本表设计被解释变量为二值变量即高发区或低发区，因此建立二项*Logistic*回归模型探讨各个解释变量如燃煤种类和各种工厂的兴建对不同区域的影响。采用极大似然估计法对回归方程进行检验。调查问卷中的各个哑变量的确定及其赋值情况详见表 3，燃料类型以木柴为参照类，附近无工厂作为另三项的参照类，其它定距变量均用原始调查表中该项数值直接代入方程。

**3:** 编码类目型变量表 Variable table of coding method

Item		Frequency	Parameter coding		
			1	2	3
Fuel type	Smoky coal	156	1	0	0
	Coke	22	0	1	0
	Smokeless coal	25	0	0	1
	Wood	77	0	0	0
Coking plant	Yes	113	1	-	-
	No	167	0	-	-
Metal smelting	Yes	83	1	-	-
	No	197	0	-	-
Chemical plant	Yes	30	1	-	-
	No	250	0	-	-

使用统计软件对表中数据进行分析时，采用向前逐步筛选策略，表 3中的变量如炼焦、金属冶炼和化工厂均没有进入方程，只有燃料类型进入到最终的筛选结果中（表 4）。以使用木柴为参照，烟煤和焦煤的回归系数β值为正值，提示烟煤和焦煤的使用与肺癌高发具有正关联作用，Exp(β) > 1，且Wald观测值所对应的概率*P*均 < 0.05，说明烟煤和焦煤较不产煤对于*Logit P*平均增长2.162和1.855个单位，结合Exp(β)值可知使用烟煤和焦煤在高发区的发生比是木柴的8.689倍和6.389倍。无烟煤的回归系数β值为-1.952，提示木柴、无烟煤与肺癌高发具有负关联作用，Wald观测值所对应的概率*P* < 0.05，且Exp(β) < 1，说明无烟煤在高发区的发生可能性与烟煤和焦煤相反。二项*Logistic*回归分析的结果表明，当地居民使用燃料类型是影响肺癌高发的重要因素，其中烟煤和焦煤对当地肺癌高发的有促进作用，而无烟煤或者木柴作用不大或有抑制作用。

**4:** 环境状况调查问卷的二项*Logistic*回归分析（逐步筛选策略） Binary *logistic* regression analysis of environmental survey by means of stepwise strategy

Fuel	*β*	SE	Wald	df	Sig.	Exp(*β*)	95%CI for Exp(*β*)	
							Lower	Upper
Wood	-	-	54.201	3	< 0.001	-	-	-
Smoky coal	2.162	0.428	25.495	1	< 0.001	8.689	3.754	20.111
Coke	1.855	0.778	5.678	1	0.017	6.389	1.390	29.369
Smokeless coal	-1.952	0.567	11.862	1	0.001	0.142	0.047	0.431

## 3 讨论

本次流行病调查调查抽样框架合理，调查点严格按设计要求抽取，调查点分布基本与曲靖地区煤炭资源分布一致，调查区域体现了肺癌高、低发区的特点，保证了设计、抽样的科学性和样本的代表性。在流行病学调查的全过程，均采取严格的质量保证和质量控制措施来确保数据的准确性和可靠性。自然村环境污染状况的调查问卷的统计分析结果表明，使用的燃料类型是当地肺癌高、低发区形成的关键因素，其中烟煤和焦煤对当地成为肺癌高发区的影响较大，而无烟煤的作用则与之相反。金属冶炼、化工生产、大规模炼焦等企业在高发区要高于低发区，虽然对环境有一定影响，但大多数生产时间较短，且就目前情况来说对当地肺癌的影响尚不明显。

附表见http://www.lungca.org/files/survey0308.pdf
